# A Molecular Survey of *Rickettsia felis* in Fleas from Cats and Dogs in Sicily (Southern Italy)

**DOI:** 10.1371/journal.pone.0106820

**Published:** 2014-09-09

**Authors:** Elisabetta Giudice, Simona Di Pietro, Antonio Alaimo, Valeria Blanda, Rossella Lelli, Francesco Francaviglia, Santo Caracappa, Alessandra Torina

**Affiliations:** 1 Department of Veterinary Sciences, University of Messina, Messina, Italy; 2 Istituto Zooprofilattico Sperimentale della Sicilia “A. Mirri”, Palermo, Italy; 3 Local Animal Health Veterinarian, ASP (Azienda Sanitaria Provinciale) Palermo, Palermo, Italy; The University of Hong Kong, Hong Kong

## Abstract

*Rickettsia felis*, the agent of flea-borne spotted fever, has a cosmopolitan distribution. Its pathogenic role in humans has been demonstrated through molecular and serologic tests in several cases. The cat flea (*Ctenocephalides felis*) is considered the main reservoir and the biological vector. The aim of this study was to assess the presence and occurrence of *R. felis* in fleas collected from dogs and cats in various sites of Palermo (Sicily). Between August and October 2012, 134 fleas were collected from 42 animals: 37 fleas from 13 dogs and 97 fleas from 29 cats. Two species of fleas were identified: 132 *Ctenocephalides felis* (98.51%) collected on all animals and only two *C. canis* (1.49%) on one dog. Out of 132 *C. felis*, 34 (25.76%), 12 from dogs (32.43%) and 22 (22.68%) from cats, were positive for *R. felis* DNA by a polymerase chain reaction (PCR), confirmed by sequencing. The only two *C. canis* fleas were negative. About half of examined animals (47.62%, 20/42) were infested with at least one infected flea; in particular 46.15% of dogs (6/13) and 48.28% of cats (14/29). It seems that in the Palermo district there is a peri-domestic cycle, with a relatively high prevalence of *R. felis* infection in the cat flea, an insect widely diffused in home environments and which can frequently bite humans. The results also suggest that *R. felis* should be considered in the human differential diagnosis of any spotted-like fever or febrile illness without a clear source of infection in Sicily, especially if the patient is known to have been exposed to flea bites.

## Introduction

Rickettsioses are vector-borne zoonotic infections caused by obligate intracellular bacteria of the genus *Rickettsia*.

*Rickettsia felis* was probably first detected in *Ctenocephalides felis* in 1918, and named “*Rickettsia ctenocephali*” [1]. However, this record was neglected until 1990, when a *Rickettsia*-like organism was found in *C. felis* fleas by electron microscopy [2]. At that time, it was referred to as the “ELB agent”, for the original source of the fleas at Elward Laboratory (Soquel, CA, USA). The species *R. felis* was formally validated by molecular criteria in 2001, and the reference strain was isolated and definitely characterized in 2002 [3]. *R. felis* has recently been included in the rickettsial transitional group [4].

*R. felis* is the etiological agent of flea-borne spotted fever (also known as cat flea typhus), described recently as an emerging rickettsiosis of medical importance [5]. Its pathogenic role in humans has been demonstrated through PCR and serology in several cases, prevalently described in hot countries [6]. Typically, the disease presents as a flu-like acute febrile syndrome, very similar to murine typhus [7], associated with headache and rash. Other signs include: asthenia, myalgia, local lymphadenopathy, neurological signs, conjunctivitis, gastrointestinal involvement and cutaneous manifestations, such as generalized maculo-papular exanthema, and in some cases a characteristic inoculation eschar at the site of the flea bite; no fatalities have been reported [5],[8]–[10]. Due to non-specific febrile clinical course, it is thought that many human cases are currently misdiagnosed as other rickettsial and viral infections. In recent years, *R. felis* has acquired an important role in the etiology of the acute febrile syndrome in many countries [6],[11]. Several cases originally diagnosed as other rickettsial infections, in particular murine typhus, were retrospectively re-evaluated by molecular or serological tools as *R. felis* infections [6],[12]–[14].

Although only a few confirmed human cases have been described, *R. felis* is globally distributed and it is primarily associated with cat fleas, *Ctenocephalides felis*, which appears to be its main vector and reservoir and the only known biological vector. It is also harbored by a variety of hematophagous arthropods [5],[6],[15]–[19].

*R. felis* transmission is primarily vertical (trans-ovarial and trans-stadial) within a flea population, rather than horizontal between fleas through a bloodmeal [20],[21]. Although not required for the maintenance of the enzootic cycle, mammalian hosts still serve as a mechanical vehicle and bloodmeal source to support flea populations. Within the flea, *R. felis* infection is disseminated, having been identified by PCR and microscopy in several tissues [22].

The wide distribution of *R. felis* is related to the worldwide distribution of *C. felis*. The cat flea is extremely common on cats and dogs in many temperate and tropical regions, but it can also infest other animal species and humans [23]. It represents the great majority of fleas in human homes.

*R. felis* has now been described in infected arthropods from more than 20 countries all over the world, excepting Antartica [5],[6],[15],[17],[24].

In Italy, only a few studies have been carried out to establish the distribution of *R. felis* infection in invertebrate hosts [25]–[27], and no clinical case or infection has yet been reported in humans or other mammals.

The aim of our study was to determine the presence and occurrence of this pathogen in fleas collected from dogs and cats in various sites of Palermo (Sicily, Southern Italy).

## Materials And Methods

### Geographical Area

The study was conducted in the city of Palermo (North coast of Sicily, Southern Italy), between August and October 2012 (temperature: mean value 24,6°C, T min: 18,3°C, T max: 30,8°C; humidity: mean value 66,3%, min 61,8%, max 71,6%).

### Flea Collection And Identification

The study was carried out on 42 flea-infested animals, 13 dogs and 29 cats.

Both dogs and cats came from various districts of the urban area of Palermo; they were primarily enrolled at the municipal shelter, but also in private veterinary facilities. Among these, 39 were strays and 3 (2 cats and 1 dog) client-owned animals.

The stray cats and dogs were captured by municipal shelter personnel or by authorized volunteers from animal protection associations, as part of a routine procedure for heath and reproductive control. The cats were captured in special cages and the dogs with painless systems and without the use of leghold traps, poisoned morsels or prods. To recover the fleas, the animals were combed craniocaudally with a plastic fine-toothed flea-comb for at least 15 minutes on the dorsal and ventral trunk. In the stray animals, this was performed after sedation with tiletamine-zolazepam (Zoletil, Virbac) prior to sterilization surgery.

For each animal, the fleas were harvested, kept alive for a few days at room temperature to allow the insects to cleanse themselves of any ingested blood, rinsed with distilled water and stored in 70% ethyl alcohol at room temperature.

The fleas were identified according to previously described identification keys [28],[29].

### Dna Extraction From Fleas

After identification, samples of fleas collected from each animal and containing more than one flea were pooled, according to species and sex. Pools ranged from two to six specimens; fleas were grouped in further pools when more than six were collected from the same animal.

The fleas were sectioned longitudinally and one half of each exemplar was immediately subjected to molecular DNA analysis. The other half of each flea was maintained in alcohol, awaiting further analysis. Two specific solutions (180 µL of Genomic Digestion Buffer and 20 µL of Proteinase K) were used overnight, until complete dissolution of flea tissues.

DNA was extracted from each pool using the PureLink Genomic DNA kit (Invitrogen by ©2012 *Life* Technologies Corporation, Carlsbad, CA, USA) following the manufacturer's instructions. In the case of a positive result in a pool, DNA was extracted from the other half of every single flea present in the positive pool and individually subjected to amplification by polymerase chain reaction (PCR), to evaluate the number of positive fleas in each pool.

### Molecular Analysis

The extracted nucleic acids were analyzed to detect the presence of DNA from *Rickettsia* spp. PCR targeting a *17 KDa* gene region [30] was performed using GoTaq Polymerase (Promega, Madison, WI, USA). For each reaction, a positive control, consisting of DNA extracted from *Rickettsia conorii* Malish 7 cultured in VERO cells, and a negative control, in which DNA was replaced by Nuclease-free water (Promega, Madison, WI, USA), were used.

The fleas which were positive for *Rickettsia* spp. at the first screening were subjected to PCR amplification of the *ompA* [31], *ompB* [32] and *gltA* genes [33] to identify the *Rickettsia* species, using a multigene assay.

Electrophoretic migration of PCR products on 1.5% agarose gel containing 10 µL/mL ethidium bromide was performed to detect the presence of the expected amplicons (246 bp for *17 kDa*, 425 bp for *ompB*, 532 bp for *ompA* and 381 bp for *gltA*).

PCR products obtained by amplifying the *Rickettsia 17 KDa*, *ompA* and *ompB* genes were purified using the Wizard SV Gel and PCR Clean-up System (Promega, Italy), quantified and sent for sequencing (© 2014 Macrogen Inc., Macrogen Europe, Amsterdam, The Netherlands). The sequences obtained were aligned using Bioedit software (Tom Hall, Ibis Biosciences, Carlsbad, CA, USA) and ClustalW 2.0.10 [34] and analyzed for nucleotide sequence identity by comparing them with the ones present in the GenBank sequence database – provided by the National Center for Biotechnology Information (NCBI) – by means of the Basic Local Alignment Search Tool (BLAST).

### Statistical Analysis

An unpaired t-test was applied to compare the differences in average numbers of fleas infesting animals per month and a Pearson Chi square test was applied to assess the correlation between infected and non-infected fleas in host species (cats and dogs). The software application SPSS statistical package (SPSS Inc., USA) for Windows was used.

### Ethics Statement

This study and the procedures when employed the animals were approved by the “Ethics Committee” of the *Istituto Zooprofilattico Sperimentale (IZS) della Sicilia “A. Mirri”*.

The research was conducted on both stray and owned dogs and cats.

All treatments, housing and animal care reported in this study were carried out in accordance with the Companion Animals Protection and Prevention of straying animals Law (15/2000) of the Government of Sicily, based on the EU Directive 2010/63/EU for animal experiments.

Pet owners gave their consent to having their animals involved in this study.

## Results

### Flea Collection And Identification

A total of 134 fleas were collected from all the animals: 37 fleas from dogs and 97 from cats. The number of fleas varied from 1 to 20 on each animal (3.2±3.6 fleas), with no significant differences (t-test: p>0.05) in average values for month: 3.0±4.1 flea/animal in August, 3.7±3.4 in September and 2.5±1.3 in October.

Two species of flea were identified: 132 (98.51%) belonged to *Ctenocephalides felis* and only two (1.49%) belonged to *Ctenocephalides canis*. *C. felis* fleas were collected on all the animals and were 29 males and 103 females, while *C. canis* fleas, one male and one female, were collected on one dog.

### Molecular Analysis Of Fleas

**Figure 1:**
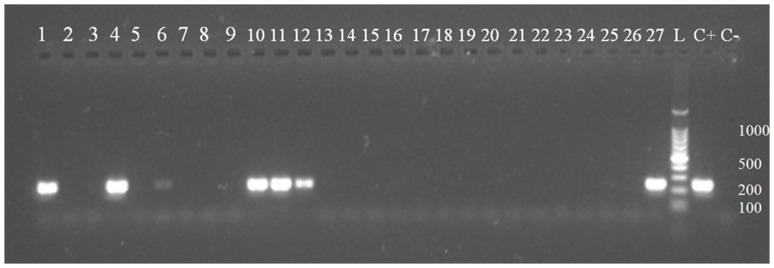
Electrophoresis on 1.5% agarose gel of the *Rickettsia* spp. PCR (246 bp). Lines 1–27: DNA from flea samples. Lane L: 100 bp ladder. Lane C+: positive control. Lane C-: negative control.

Out of the 132 *C. felis*, 34 (25.76%) were positive for *Rickettsia* spp. at the *17 kDa* PCR (Fig. 1). All of these 34 fleas were also positive at the *ompB* PCR and 20 of them at the *ompA* PCR. No fleas were positive at the *gltA* PCR. The two specimens of *C. canis* were negative and collected from one dog. The same dog also harbored four cat fleas, also negative for *Rickettsia* spp. DNA. A total of 12 positive fleas (32.43%) were collected from dogs and 22 (22.68%) from cats (Tables 1, 2). Although the positivity was higher in fleas taken from dogs than in those taken from cats, the statistical analysis revealed no significant differences between the two populations (chi-square  = 1.29).

**Table 1:** Flea distribution on dogs (^§^owned dog), with the results of *Rickettsia* spp. PCR performed on 37 fleas (*two *C. canis*).

Dog Id. Nr.	Fleas		
	Nr.	*Rickettsia* spp. PCR (*17 kDa*)	
		+	−
1	1	0	1
2	1	0	1
3	3	0	3
4	6*	0	6
5	1	0	1
6	3	2	1
7	3	2	1
8	4	3	1
9	3	0	3
10	4	1	3
11	4	1	3
12	3	3	0
13^§^	1	0	1
**Total**	**37**	**12 (32.43%)**	**25 (67.57)**

**Table 2:** Flea distribution on cats (^§^owned cat), with the results of *Rickettsia* spp. PCR performed on 97 fleas.

Cat Id. Nr.	Fleas		
	Nr.	*Rickettsia* spp. PCR (*17 kDa*)	
		+	−
1	1	1	0
2	2	0	2
3	1	1	0
4	1	0	1
5	1	0	1
6	1	0	1
7	3	3	0
8	1	0	1
9	6	0	6
10	4	0	4
11	1	1	0
12	1	0	1
13	2	0	2
14	4	2	2
15	1	1	0
16	3	3	0
17	1	1	0
18	1	0	1
19	13	0	13
20	11	1	10
21	20	0	20
22	3	1	2
23	2	0	2
34	2	1	1
25	2	0	2
26	3	3	0
27	2	2	0
28	2	1	1
29	2	0	2
**Total**	**97**	**22 (22.68%)**	**75 (77.32%)**

The positive insects were collected from 20 out of 42 animals (47.62%), 6 dogs (46.15%) and 14 cats (48.28%). All the animals with positive fleas except one (a cat) were strays.

In order to identify the *Rickettsia* species, 31 out of 34 PCR products were sequenced. For the other three samples the starting material was not of sufficient quantity to proceed with sequencing. Obtained sequences were compared with those deposited in GenBank and all of them showed a very high degree of similarity to *R. felis* sequences. All the sequences found in this study were submitted to GenBank with the following Accession Numbers: BankIt1735386: KM006831–KM006861 for *17 kDa* sequences; BankIt1733537: KM006812–KM006830 for *ompB* sequences and BankIt1733287: KM006781–KM006811 for *ompA* sequences (Tables 3, 4).

**Table 3:** Sequencing results for *Rickettsia* spp. positive *C. felis* collected from dogs.

Dog Id. Nr.	*Rickettsia felis*					
	*17 kDa*		*ompB*		*ompA*	
	A.N. BankIt1735386	Reference A.N. (% identity)	A.N. BankIt1733537	Reference A.N (% identity)	A.N. BankIt1733287	Reference A.N. (% identity)
**6**	KM006833	DQ102709.1 (99%)	KM006802	GQ385243.1 (99%)		
**6**	KM006845	DQ102709.1 (99%)	KM006798	GQ385243.1 (93%)	KM006829	JN990593.1 (98%)
**7**	KM006846	DQ102709.1 (99%)	KM006794	GQ385243.1 (99%)	KM006823	JN990593.1 (98%)
**7**	KM006849	DQ102709.1 (97%)	KM006793	GQ385243.1 (99%)		
**8**	KM006840	DQ102709.1 (99%)	KM006797	GQ385243.1 (97%)	KM006824	AJ563398.1 (99%)
**8**	KM006850	DQ102709.1 (97%)	KM006808	GQ385243.1 (94%)		
**8**	KM006851	DQ102709.1 (98%)	KM006807	GQ385243.1 (100%)	KM006822	AJ563398.1 (99%)
**10**	KM006852	DQ102709.1 (99%)	KM006806	GQ385243.1 (100%)		
**11**	KM006855	DQ102709.1 (98%)	KM006788	GQ385243.1 (99%)	KM006827	AJ563398.1 (95%)
**12**	KM006856	DQ102709.1 (98%)	KM006787	GQ385243.1 (100%)	KM006818	JN990593.1 (94%)
**12**	KM006838	DQ102709.1 (98%)	KM006792	GQ385243.1 (94%)	KM006815	JN990593.1 (94%)
**12**	KM006839	DQ102709.1 (98%)	KM006795	GQ385243.1 (95%)		JN990593.1 (94%)

For each gene amplified and sequenced (*17 kDa*, *ompB* and *ompA*), the Accession Number (A.N.) of the corresponding sequence submitted in GenBank and the percentage (%) of identity with respect to reference sequence of *R. felis* present in GenBank are reported.

**Table 4:** Sequencing results for *Rickettsia* positive fleas *C. felis* collected from cats.

Cat Id. Nr.	*Rickettsia felis*					
	*17 kDa*		*ompB*		*ompA*	
	A.N. BankIt1735386	Reference A.N. (% identity)	A.N. BankIt1733537	Reference A.N (% identity)	A.N. BankIt1733287	Reference A.N. (% identity)
1	KM006831	DQ102709.1 (100%)	KM006781	GQ385243.1 (99%)		
3	KM006832	DQ102709.1 (99%)	KM006784	GQ385243.1 (100%)	KM006812	JN990593.1 (99%)
7	KM006860	DQ102709.1 (100%)	KM006785	GQ385243.1 (100%)	KM006830	AJ563398.1 (99%)
7	KM006843	DQ102709.1 (99%)	KM006786	GQ385243.1 (99%)	KM006817	AJ563398.1 (98%)
7	KM006861	DQ102709.1 (99%)	KM006783	GQ385243.1 (99%)	KM006813	JN990593.1 (99%)
11	KM006841	DQ102709.1 (100%)	KM006782	GQ385243.1 (100%)	KM006814	AJ563398.1 (98%)
14	KM006859	DQ102709.1 (97%)	KM006799	GQ385243.1 (100%)		
14	KM006847	DQ102709.1 (100%)	KM006811	GQ385243.1 (93%)	KM006816	AJ563398.1 (99%)
15	KM006844	DQ102709.1 (100%)	KM006791	GQ385243.1 (97%)		
16	KM006858	DQ102709.1 (97%)	KM006796	GQ385243.1 (94%)		
16	Not enough					
16	KM006842	DQ102709.1 (100%)	KM006809	GQ385243.1 (99%)	KM006828	HM636635.1 (94%)
17	Not enough					
20	Not enough					
22	KM006848	DQ102709.1 (99%)	KM006810	GQ385243.1 (99%)		
24	KM006834	DQ102709.1 (97%)	KM006805	GQ385243.1 (99%)	KM006826	JN990593.1 (98%)
26	KM006857	DQ102709.1 (97%)	KM006804	GQ385243.1 (100%)	KM006821	AJ563398.1 (100%)
26	KM006835	DQ102709.1 (98%)	KM006803	GQ385243.1 (100%)	KM006819	AJ563398.1 (99%)
26	KM006853	DQ102709.1 (98%)	KM006790	GQ385243.1 (100%)	KM006820	JN990593.1 (100%)
27	KM006854	DQ102709.1 (99%)	KM006789	GQ385243.1 (100%)	KM006825	AJ563398.1 (99%)
27	KM006836	DQ102709.1 (97%)	KM006801	GQ385243.1 (100%)		
28	KM006837	DQ102709.1 (99%)	KM006800	GQ385243.1 (100%)		

For each gene amplified and sequenced (*17 kDa*, *ompB* and *ompA*), the Accession Number (A.N.) of the corresponding sequence submitted in GenBank and the percentage (%) of identity with respect to reference sequence of *R. felis* present in GenBank are reported.

## Discussion

The results of our research show that almost all fleas collected both on cats and dogs (132/134) were *Ctenocephalides felis*. This confirms the wider diffusion of the cat flea also in dogs, as previously observed in other studies carried out in Italy and in other countries [25],[35]. Although the two species of *Ctenocephalides* (*C. canis* and *C. felis*) are present throughout the world, the cat flea seems to have a broader distribution than that of the dog [23]. Both species can infest dogs and cats but also other hosts, including humans, especially during the warm months.

In our research we have observed *Rickettsia felis* infection in 25.37% of the cat fleas, confirming the frequent association between *C. felis* and the pathogen. The positivity is higher in fleas taken from dogs compared to those taken from cats, although without significant differences.

Although the sample size is small, particularly in the number of dogs and cats enrolled, our data make a contribution to the understanding of the spread of infection not only in Sicily, where information on this matter was not previously available, but also in Italy, where only a few studies have been carried out [25].

Our results differ in part from what has been observed in a previous study carried out in other areas of Italy, where the prevalence was lower on average (11.9%), but similar in the Northeast (23.2%; 26/112 fleas). This study also recorded a higher percentage in fleas from cats (17.6%) than in fleas from dogs (10.2%) [25].

Recent surveys carried out in various European countries showed highly variable prevalence rates of the infection in *Ctenocephalides* fleas from dogs and cats: 2.76% (10/371 fleas) in Albania [36]; 17.69% (95/537 fleas) in France [37]; 20.66% (25/121 pools of fleas) in the United Kingdom [38]; 19.91% (44/221 pools) in the Netherlands [39]; 43.59% (34/78 fleas) in Catalonia [40] and 54.17% in Andalusia [41]; 8.89% (24/270 fleas) in Germany, where a high positivity in different species of fleas was also reported (100%, of *Archeopsylla erinacei*) [42].

Our finding of negativity of *C. canis* cannot lead to any conclusion (only two specimens). In our previous research carried out on fleas collected from foxes in the same area, we observed no positivity in 32 *C. canis* and in other 76 fleas of various species, while the only two *C. felis* were both positive [27]. Other authors have reported the absence [25],[41],[42] or a low prevalence [39],[40] in larger sample sizes, in contrast to what has been observed in France, where 27.03% of *C. canis* (10/37) were infected [37].

In our study, about half the animals examined (47.62%; 20/42) were infested with at least one *R. felis* infected flea; specifically 46.15% (6/13) of dogs and 48.28% (14/29) of cats. It is noteworthy that the flea specimens were almost all collected from stray dogs and cats and not from kennels or catteries, where the prevalence of *R. felis* infection is often higher [24]. Hence, it may well be that infection rates in closed animal populations are higher than those presented. Moreover, the presence of an infected flea in one of the three owned animals in our sample, a household cat, is interesting and might suggest an easier transmission to humans. In fact, the seroprevalence of individuals that have reported contact with domestic animals tends to be higher [43].

It will also be interesting to extend the research on pet hosts. The role of mammals as a reservoir of this emerging flea-borne infection needs further confirmatory evidence, because there is to date no consensus on the potential mammalian reservoir(s). The presence of *R. felis* has been recorded in several peri-domestic species associated with the cat flea, including cats, dogs, opossums and rats [5],[15],[17],[23],[35], but no clinical case has been described in animal hosts, except humans. Recently, it was suggested that the dog [35],[44] and the hedgehog [42] might play a role in the ecology of the pathogen in some areas.

This study shows a potential risk of transmission to humans in the Palermo district, at least in conditions where there is increased contact with stray animals and their ectoparasites (e.g., categories of workers, such as veterinarians, kennel or cattery personnel, volunteers employed in animal capture, etc.).

Although human cases of flea-borne spotted fever are yet to be reported in Italy, it would seem that this infection is more common than is currently recognized. In fact, clinical symptoms are similar to those of other rickettsial diseases (e.g., murine typhus, MSF), making a causal diagnosis difficult. Because antigenic cross-reactivity exists among different species of rickettsiae [14], serological assays are likely to be insufficient to definitively identify *R. felis* unless other, more sophisticated, serological (Western blot and cross-absorption studies) or molecular assays are performed [6]. Given our findings, we cannot exclude the possibility that *R. felis* could be implicated in Mediterranean spotted fever, endemic in Southern Italy (Sicily alone accounts more than half of all the national clinical cases) [45]–[47], or in murine typhus due to *R. typhi*, sporadically reported in Sicily [45]. In fact, diagnosis of MSF in Italy usually depends on clinical evidence supported by serologic confirmation. Current diagnostic tests, however, use *R. conorii* as the only antigen [47] and hence, due to possible cross-reactions, misdiagnosis may occur.

In light of the above, we conclude that flea-borne spotted fever should be included in the human differential diagnosis of any spotted-like fever or febrile illness without a clear source of infection in Sicily, especially if the patient is known to have been exposed to flea bites. Moreover, careful hygiene and cleaning of pet beddings and a wider use of pesticides in domestic animals, as well as having a direct benefit to the animals themselves, could reduce the risk of transmission of pathogens dangerous to humans.
